# Infant feeding practices among HIV-positive mothers at Tembisa hospital, South Africa

**DOI:** 10.4102/phcfm.v9i1.1278

**Published:** 2017-07-27

**Authors:** Armelia Chaponda, Daniel T. Goon, Muhammad E. Hoque

**Affiliations:** 1 Department of Health Studies, University of South Africa, South Africa; 2 Department of Nursing Science, Faculty of Health Sciences, University of Fort Hare, South Africa; 3 Graduate School of Business and Leadership, University of KwaZulu-Natal, South Africa

## Abstract

**Background:**

Despite the nutritional, physiological and emotional benefits of breastfeeding, HIV-positive mothers cannot practise exclusive breastfeeding for six months because of a range of influences on their feeding choice – thereby creating a caveat for morbidity in infants.

**Aim:**

This study explored factors influencing the infant feeding choice of HIV-positive mothers at a peri-urban hospital in Tembisa, South Africa.

**Methods:**

This study was qualitative and was conducted among 30 purposefully selected postnatal HIV-positive mothers at Tembisa hospital, Gauteng, from May to June 2011. In-depth interviews were conducted mainly in isiZulu and Sepedi which were then transcribed into English. An open coding system of analysis was used for thematic analysis.

**Results:**

Nurses significantly influenced the feeding choices of new mothers – sometimes with inconsistent information. The grandmothers of infants also influenced the new mothers’ feeding options, in some cases with the new mother coming under duress. Other relatives like the sisters and aunts of mothers appeared to significantly affect feeding choices. The time frames expressed for the initiation of a supplementary diet were as follows: before 1 month, at 1 month and at 4 months. The main reason was the belief that infants required more than breast milk as sustenance during this period.

**Conclusion:**

In the postnatal hospital setting of this study, the feeding choices of mothers were influenced by nursing personnel. Nursing personnel could marry the influential ‘authority’ they have with correct and consistent information, in order to change feeding behaviour. Significant ‘others’ like grandmothers and other relatives also influenced decisions on infant feeding. As such, family dynamics need to be considered when encouraging breastfeeding.

## Introduction

The benefits of breastfeeding are incomparable and comprise the prevention of infant infectious diseases, and reduction of leukaemia, sudden infant death syndrome, type 1 and 2 diabetes and obesity.^1^ Breast milk contains bioactive molecules that are anti-infective and anti-inflammatory.^2^ Considering the emotional affection associated with breastfeeding, for both mother and infant, it is imperative for health professionals dealing with antenatal and postnatal mothers to make all efforts to promote breastfeeding. In support of breastfeeding, the World Health Organization (WHO) recommended initiation of breastfeeding within half to one hour of birth, in order to stimulate the production of breast milk and, more importantly, colostrum, which includes nutritionally ‘protective’ factors for the infant.^3^ Even with the physiological and emotional benefits raised, breastfeeding duration rates have declined.^4^ New mothers cannot practise this recommendation because of a range of influences on their feeding choice – thereby creating a caveat for morbidity in infants, as it is influenced by the feeding choices that mothers make.^5^ In developed and developing countries, formula milk and animal milk have been associated with adverse growth and development in infants.^6,7^ In low-income countries, HIV-exposed infants have succumbed to morbidity and mortality caused by the early cessation of breastfeeding.^8^ In Malawi, the rates for hospitalisation because of gastroenteritis increased with cessation of breastfeeding at 6 months.^9^ Similarly, in Zimbabwe, infants were inadequately fed at 9 months after the cessation of breastfeeding.^10^

The ‘environment’ in which expectant and new mothers find themselves plays a significant role in the choice of feeding method. The people, economic circumstances and social circles that mothers are in contact with influence the well-being of the infant – therefore making breastfeeding a behaviour that mothers learn. Health personnel can inadvertently – because of hospital routines such as allowing the mother to rest after a long and difficult labour – hinder successful latching and duration of breastfeeding. Similarly, relatives who generally have the new mother and infant’s best interests at heart can encourage different feeding methods to those chosen by mothers. Understanding the barriers to infant breastfeeding will inform public health policy regarding the promotive strategies of breastfeeding. The choice of infant feeding is often influenced by factors other than the mother’s personal wish. The knowledge, culture and social status of caregivers also often play a significant role in infant feeding – as in rural Ghana.^11^

Difficult economic and social circumstances are often reasons why some mothers opt for a cheaper feeding option or why they resume breastfeeding after alternative foods are introduced – thus effectively mix-feeding their infants. Most families cannot afford to purchase, prepare or properly store infant formula. Breast milk substitutes require careful measurement, preparation and storage under sterile conditions, which is often difficult or impossible in many African social and economic situations – especially in poor communities. In circumstances where the provision of infant formula is unsustainable, families resort to home foods which are largely poorer in nutritional quality, and they have deficits in terms of energy, vitamin A, iron, calcium, zinc and other essential nutrients.^12^ Morbidity in infants is influenced by the feeding choices that mothers make.^5^ Supplementary foods damage the intestinal lining of newborn babies, making them more susceptible to HIV infection.^13^ Only 25% of mothers in South Africa practise exclusive breastfeeding, while 75% use formula for feeds during the first six months.^14^ In-depth understanding of the influences on feeding decisions is required for optimum support of the adoption of the WHO guidelines on HIV and infant feeding, and for new mothers who wish to breastfeed. Considering the above background, this study explored factors influencing infant feeding choice at a peri-urban hospital in Tembisa, Gauteng, South Africa.

## Methodology

### Study design and study setting

A qualitative, explorative design was used to enable sensitivity to context. The study was conducted in Tembisa hospital. This hospital is a regional hospital in the Northern Service Delivery Region in Ekurhuleni, Gauteng. The two wards admitted mothers – and infants who were delivered either via spontaneous vertex delivery (SVD) or via caesarean section (C/S) – for observation and management of possible puerperal complications prior to discharge. The daily number of SVD and C/S was 20–30. Tembisa hospital provided formula for infants in the postnatal wards, as part of the national prevention of mother to child transmission (PMTCT) strategy. On discharge from the postnatal ward, a family-planning nurse provided health education on contraception and infant feeding: primarily breastfeeding and its contraceptive qualities, if done exclusively.

### Sample selection and data collection

A total of 30 postnatal HIV-positive mothers were purposefully selected for the study from two postnatal wards at Tembisa hospital. HIV-positive mothers over 18 years old, and residing in Tembisa, were sampled. Participation was not dependent on feeding choice, nor on being on antiretroviral (ARV) therapy and participating in PMTCT programmes. In-depth interviews were conducted during May and June 2011. Data gathering continued until data saturation occurred – when no further new information could be obtained. The interviews were recorded using a tape recorder. Also, while individual interviews were conducted, the postnatal mothers were observed in the postnatal ward while breastfeeding their infants.

### Data analysis

After all in-depth interviews were tape-recorded, a full transcript of the discussion was prepared. The discussion completely and as accurately as possible reflected the participants’ own words. The key statements and ideas expressed were listed. After transcripts of the discussion were prepared, the participants’ statements were coded. Finer sub-codes were used to capture as much as possible. The data were captured into a Microsoft Excel spreadsheet for ease of analysis and for analysis using an open coding system. This method reduced the data to themes, categories and sub-categories.

## Results

Through thematic content analysis, the four identified themes were identified with regard to the following: influential person on their feeding choice, their chosen feeding choice, their concerns with breastfeeding and their initiation of a supplementary diet.

### 

#### Theme 1: Opinions of significant ‘others’ on feeding choice

The data indicated there are people who affect the feeding choices that mothers make. Some mothers have thought about which feeding method to use and yet were pressurised by relatives or other significant persons to use another method. Furthermore, the data suggested that some mothers used factors like cost, reliability and ease of use, when deciding on the mode of feeding. These mothers reported that people they reside with, as well as professional nurses, influenced their feeding choice.

#### Sub-theme 1.1: Nurses have an influence over mothers’ feeding decisions

It was found that the nurses are considered dominant in the postnatal ward setting and swayed feeding choices to their preference. Additionally, mothers were ‘told’ what to do and were shouted at when they were not conforming.

‘Me … I will have to say it’s the nurses. They tell us what to do with our babies and they are not happy if we do not do as they say. Sometimes you go there and if they find out you are not doing what they told you to do, then they shout at you.’ (Participant B9, female, 27 years)

‘That nurse, she said I must do like that. So you must do it – you must put the baby to your breast.’ (Participant B26, female, 31 years)

#### Sub-theme 1.2: Relatives have a significant influence on feeding decisions

Mothers lived mostly with their relatives and reported that those relatives also had a significant influence on their feeding decisions – and so much that they felt they had to adopt the proposed method. These relatives are sisters and aunts who may or may not have children of their own. The data indicated these influences can be worrying, causing some degree of anguish.

‘The people I stay with. They are my relatives. They always tell me what to do with my children. Even the last babies. Now with this one, I don’t know.’ (Participant B23, female, 34 years)

‘My sister, she had a baby, now with mine she is going to tell me what to do with the baby.’ (Participant B7, female, 29 years)

#### Sub-theme 1.3: Grandmothers’ influence on feeding decisions

Grandmothers are instrumental in shaping feeding decisions at household level. This is partly because they become the primary caregiver after the birth of their grandchildren. In this study, negative past experiences informed grandmothers’ decisions – which they then impose on new mothers. The data suggest that some mothers are persuaded into choosing a feeding option proposed by their mothers, even when they are uncomfortable with that decision.

‘Yes at home … it is my mother. At the beginning, the first baby she saw was the one that died. I breastfed the baby and now she is saying that I should not breastfeed this one. I don’t have money for milk. [*Researcher: Does your mother know that you are HIV-positive?*] No, she does not have that information.’ (Participant B10, female, 30 years)

‘I stay with my mother now and she is taking care of me and the baby. She said I must feed the baby by breast [*Researcher: what do you say?*] I don’t know, I will join her.’ (Participant B4, female, 20 years)

### Theme 2: Infant feeding choice

New mothers were asked which feeding option they preferred to use with their new infant, when comparing breastfeeding with infant formula and the flash-heat technique (heat treating expressed breast milk).

#### Sub-theme 2.1: Infant formula

At most 15 mothers believed that the use of infant formula resulted in better health outcomes compared to the other methods. The data showed that mothers believed that breast milk will be insufficient in terms of satisfying their infants.

‘I am going to formula-feed this baby. I was going to buy Parlagon to feed my baby. Parlagon makes babies grow well, [*and*] that is why the hospital uses it. They don’t get sick easily. I did not breastfeed my first baby and that’s why I am not going to breastfeed this baby.’ (Participant B3, female, 37 years)

#### Sub-theme 2.2: Breastfeeding

The other half of mothers (15) breastfed their infants. The mothers mentioned that they witnessed other mothers breastfeeding their infants and that is why they decided to do the same. In most cases, this person would be someone they lived with.

‘I will give my child the breast. In my family we do that. So I will also do that to my child.’ (Participant B24, female, 32 years)

#### Sub-sub-theme 2.2.1: Concerns with breastfeeding

When the mothers were asked why they did not choose to breastfeed their infants, they said they believed they could not do so because of insufficient breast milk. What mattered more to them was that the quantity would be insufficient for feeding; the nutritional value that breast milk has over the alternative was considered less important.

‘For me … I don’t have enough milk in my breasts. That is my problem. Even with my first daughter, I did not have enough milk. So I will just go and buy Nan formula. But If I had milk, I would follow breastfeeding because it is good.’ (Participant B18, female, 26 years)

‘I give my baby powder milk. I think it is better, because the baby will get enough. He will cry for more milk if I give him my breast.’ (Participant B8, female, 31 years)

The other doubt raised was the duration of breastfeeding.

‘The thing with breastfeeding is the question of how long a person should breastfeed for.’ (Participant B21, female, 29 years)

### Theme 3: Initiation of a supplementary diet

The data indicated that most mothers had very different views on the timing of complementary feeding. Some believed in introducing complementary feeding at 1 month, whereas others believed it should be introduced at 4 months. An interesting finding is giving a baby medicine to ‘clean’ its bowel after birth – this being information they received from relatives.

‘I think at 1 month. My mother and sister I stay with say [*that*] I must wait for 1 month before I feed anything, then after 1 month I can give porridge to the baby.’ (Participant B4, female, 20 years)

‘For me I think 4 months. I see the baby he gets food at 4 months, not only the milk but other food like tea and porridge. The milk from the breast is not enough food for the baby.’ (Participant B6, female, 25 years)

‘You know us Black people have to give something to the babies to clean them before 1 month. Clean the stomach from the blood of the mother – the baby eats this blood when it is not yet born.’ (Participant B10, female, 30 years)

### Theme 4: Counselling on infant feeding

The information mothers recall receiving was inconsistent and sometimes was not provided. The correct breastfeeding period appeared to elude the mothers. Furthermore, a few mothers reported not receiving any information from professional nurses on the best timing for the introduction of supplementary foods.

‘The nurses did not tell me anything about how long to feed my baby on this breast milk.’ (Participant B4, female, 20 years)

‘My sister put her baby to the breast for 3 months. That baby is walking today and is very strong. My baby is also going to do that, because I will feed her for the same. [*Researcher: What did the nurse tell you about breastfeeding?*] Yes I think she said 4 to 6 months, I am sure.’ (Participant B24, female, 32 years)

‘I don’t know, maybe they told me, but I don’t remember.’ (Participant B12, female, 24 years)

## Discussion

The results of this study entailed four themes relating to new mothers: influential people affecting feeding choice, chosen feeding choice, concerns with breastfeeding and initiation of a supplementary diet.

### Opinions of significant ‘others’ on feeding choice

In this study, most participants reported that relatives played a significant role in shaping their decisions about what to feed their infants. This is similar to the findings of another study where the mother-in-law saw her responsibility as making decisions about the infant’s health, when the mother moved to her daughter’s home shortly after the birth.^15^ Similarly, in this study, the infants’ grandmothers had a significant influence on feeding choice, and this is corroborated by older relatives in a different study who also influenced new mothers with regard to infant feeding.^16^

Nurses were also influential in terms of affecting feeding decisions, and this confirmed their high power and influence over the feeding choices of mothers.^17^ The power dynamics of the nurse–patient relationship come to the fore where the nurse becomes a ‘power figure’ and the patient or new mother a ‘recipient’ of health advice and not an active participant in health decisions. The power dynamics also emerged in a different study, where Tanzanian nurses felt that antenatal mothers expected to be told which feeding method was best. Nurses were considered to be ignorant of their work if they did not exercise that ‘authority’.^18^ The same study reported that nurses could not adapt to counselling, which was seen as allowing mothers to decide which feeding method was optimum – even when they made the incorrect choices.^18^ This is fundamental in understanding counselling behaviour and requires a re-examination of counselling training for public health sector nurses. Another study found that healthcare workers and counsellors (which nurses fall under) were not the most influential in terms of infant feeding matters – making infant feeding contentious.^16^

This study found that mothers felt pressurised into using a feeding method chosen by relatives and reported feeling helpless in terms of protecting their infants from HIV infection – primarily because they had not disclosed their status to the significant ‘other’. The advice given to mothers is termed ‘blind advice’; an advisor is typically unaware of HIV status.^16^ Disclosure is significant in facilitating understanding of the choice of feeding, and creates an environment conducive to discussions on matters affecting health.

Additionally, most participants were ‘told’ by nurses which feeding method to choose – and they felt compelled to follow that option. The culture of ‘telling’ mothers what feeding option to choose may impact on child health, development and survival, as mothers feel pressured to follow that ‘advice’ – even amidst financial and economic difficulties. Furthermore, ‘telling’ does not characterise choice-making on infant feeding. The researchers confirmed this when HIV-positive mothers in Khayelitsha were not allowed any choice in terms of infant feeding.^19^ Other researchers concluded that nurses in KwaZulu-Natal made mothers feel guilty for exercising their own judgement in terms of feeding options.^20^

### Poor infant feeding counselling

New mothers made feeding decisions based on the information they received from their relatives and nursing personnel. Nursing personnel are trained to provide correct, evidence-based education to new and recurring mothers alike, and yet fail in their obligations to do so. Relatives mostly do not have the training to educate on feeding choice, but nonetheless feel that they are equipped to do so. The result is an outcome where a sizeable number of the infants in this study are not breastfed. The quality of the counselling based on the accuracy of information was not assessed, but can be deduced from the outcome of the mothers’ feeding choice.^21^ In a different study, the quality of counselling in South Africa through structured observations and exit interviews in three PMTCT pilot sites was evaluated. It was found that counsellors did not use a comprehensive assessment of a woman’s social and economic resources for implementing various feeding options. Instead, counselling on infant feeding was based purely on the mother’s HIV status.^22^ In another study, social pressure from relatives or nursing personnel was a significant factor in the choice of a feeding decision.^23^

### Initiation of a supplementary diet

Mothers held different views on the introduction of complementary feeding. A study in Uganda found that HIV-positive women appeared to make poorer infant feeding decisions compared to the general population – because of their fear of the association between breastfeeding and HIV transmission. This was not the primary concern raised in this study. The mothers’ concerns were mainly of the perceived ‘insufficiency’ of breast milk and the duration of breastfeeding. This is contrary to the beliefs of other HIV-positive mothers who considered they would definitely transmit the virus to their infant if they breastfed – and were therefore not willing to take that risk.^19^ This could indicate that the PMTCT programme at the antenatal clinic contributed to allaying mothers’ fears of transmitting HIV to their infants. The perception of having insufficient breast milk has been raised before in other studies – where South African mothers also believed that their breast milk was insufficient for the baby’s growth.^24^ The findings also suggest that less than half the mothers would mix-feed their infants, because they believed breast milk was insufficient for infant growth and development and that supplementary foods are essential.

The timing of the initiation of complementary feeding is of concern. This varied from 1 month to 4 months. This corroborates other findings where supplementary milks were introduced by 52% of South African mothers within the first month of feeding, and within 3 months, 82% of mothers had introduced other milks to their infants (they rarely practised exclusive breastfeeding after 3 months).^25,26,27^ Feeding an infant something to clean its gut appears not to be a new finding. This was also found in Rwanda. Here, infants were fed water before the milk came in – to ‘satisfy thirst’ or to ‘cleanse the digestive system’. Sometimes the water was sweetened or salt was added. After nursing was initiated, the same water was given at intervals, until the mother’s white milk came in.^28^

It should be stated that half of the mothers breastfed and they found having breast milk readily available for their infants was very comforting to them. This suggests that mothers need to be assured of the consistency and reliability of their infants’ milk supply. It has been reported that the mother who provides her own milk for her infant has absolute control over her own milk supply – and can assure her infant’s food security for the entire lactation period.^29^ These are important factors to consider when educating new mothers on the benefits of breastfeeding.

## Limitations of the study

This study was limited to HIV-positive mothers at Tembisa hospital in Gauteng and cannot be used to generalise to the wider population. The views are those of the mothers who were subject to the postnatal environment at Tembisa hospital. A larger sample size with HIV-positive mothers at different locations could have yielded more informative results.

## Conclusion

In the postnatal hospital setting of this study, the feeding choices that mothers made were influenced by nursing personnel, their own mothers and also other relatives. Some mothers believed that infant formula was good for infants, because they saw it given to other infants in hospital. Therefore, their assumptions were that it could not be harmful if used in that particular setting. Because nurses are significant influencers, they should be evaluated for consistency in terms of breastfeeding messages conveyed and the education offered to the public. For example, nurses cannot educate on the benefits of breastfeeding when infant formula is seen being provided to other infants in the same setting without any explanation for the reasons for this. Additionally, nursing personnel could marry the influential ‘authority’ they have with correct and consistent information, in order to change feeding behaviour. In addition, grandmothers and other relatives who have an influence on feeding decisions should be taught correct and consistent messages – in order to champion exclusive breastfeeding in the community.
